# A cancer disparities curriculum in a hematology/oncology fellowship program

**DOI:** 10.1186/s12909-023-04465-0

**Published:** 2023-10-17

**Authors:** Marium Husain, Muhammad Salman Faisal, Dionisia Quiroga, Audrey M Sigmund, Gregory Otterson, Alison Walker, Samilia Obeng-Gyasi, Beth Christian

**Affiliations:** 1 Division of Medical Oncology, The Ohio State University James Comprehensive Cancer Center, 1800 Cannon Ave, Suite 1300, Columbus, OH 43210 USA; 2 Department of Hematology and Medical Oncology, Roswell Park Cancer Institute, Buffalo, NY USA; 3 Division of Hematology, James Comprehensive Cancer Center, The Ohio State University, Columbus, OH USA; 4 Division of Hematology, Moffitt Cancer Center, Tampa, FL USA; 5 Division of Surgical Oncology, The Ohio State University James Comprehensive Cancer Center, Columbus, OH USA

**Keywords:** Health disparities, Curriculum, Hematology/oncology, Fellowship

## Abstract

**Background:**

After George Floyd’s murder in 2020, the Center for Disease Control and Prevention (CDC) called systemic racism a public health crisis. This health crisis is connected to the already-documented racial and socioeconomic disparities in cancer care. Ensuring hematologists and oncologists are aware of these disparities through their medical education can help to address these disparities.

**Methods:**

The authors implemented a healthcare disparities-focused curriculum in a Hematology/Oncology fellowship program during the 2020–2021 academic year at The Ohio State University Hematology/Oncology Fellowship Program. They implemented a pre- and post- survey to evaluate the efficacy of the program.

**Results:**

Fifteen fellows completed the pre-curriculum survey and 14 completed the post-survey. Before the curriculum, 12 fellows (80%) noted a “Fair” or “Good” understanding of healthcare disparities, and 6 (40%) had a “Fair” understanding of disparities in clinical trials and access to novel therapies. Fourteen fellows (93.3%) had not previously participated in a research project focused on identifying or overcoming healthcare disparities. After the curriculum, 12 (85%) fellows strongly agreed or agreed that the information presented in the curriculum was useful for training as a hematologist/oncologist. Twelve fellows (85%) noted “Agree” or “Strongly Agree” that the information presented was relevant to their practice. Eleven fellows (92%) noted that they plan to incorporate healthcare disparities into a future research or clinical project. The majority of fellows, 11 (79%) recommended that the fellowship program continue to have a formal health disparities curriculum in the future.

**Discussion/Conclusion:**

There is utility in incorporating cancer disparities education into a hematology/oncology academic curriculum. We recommend further analysis of such curricula to improve fellowship education and patient outcomes with these interventions.

## Background

Systemic racism in medicine has ranged from unethical experiments to race-based practice, resulting in poor health outcomes in minority patients compared to the white population.[1–3] This has translated into inequity in health outcomes and inferior overall outcomes in the United States (U.S.) despite the highest per capita health care expenditure.[4] These health disparities exist in cancer care and delivery, based on race, socioeconomic status, and gender [5]. A landmark study on cancer incidence, survival and risk factors demonstrated that residents of poorer U.S. counties (≥ 20% poverty rate) (had up to 20% higher cancer deaths compared to the residents of affluent counties (< 10% poverty rate) [6]. Even after adjusting for poverty, African Americans, American Indians and Asian/Pacific Islanders had worse five-year survival compared to non-Hispanic Whites [6] This led the American Cancer Society to establish abolishing racial disparities as one of their 2015 Challenge goals. A tipping point came with the murder of George Floyd due to police brutality in Minneapolis, Minnesota in May 2020. The resultant outcry led to all major medical organizations issuing public statements on health equity [7, 8].

Efforts in medical education, including from the American Medical Association (AMA), to advance health equity include implicit bias training, cultural humility and antiracism training which are promoted by some graduate medical programs [9, 10]. The New England Journal of Medicine (NEJM) has Case Studies in Social Medicine series since 2018 that addresses social determinants of health in case-based scenarios [11]. However, there are limited resources for healthcare disparities education designed specifically for hematology/oncology fellowship programs, largely institution-specific that are not standardized curricula. National organizations, such as the American Society of Clinical Oncology (ASCO) and American Society of Hematology (ASH) have produced resources and videos focusing on healthcare disparities after the murder of George Floyd, but there is no standardized curriculum for hematology/oncology trainees. In this study, we present our one-year effort establishing a cancer disparities curriculum.

## Methods

We implemented a novel, cancer disparities-focused curriculum for the Ohio State University (OSU) Hematology/Oncology Fellowship Program. This consisted of a combination of lectures, interactive didactic sessions, and small group discussions during the 2020–2021 academic year. This project was deemed exempt by the Institutional Review Board.

### Disparities curriculum

We conducted four virtual sessions (due to the COVID-19 pandemic). The first session was a panel discussion on healthcare disparities in hematology/oncology: disparities in care related to sickle cell disease, multiple myeloma, and breast cancer. The second session focused on implicit bias using modules developed by the OSU Kirwan Institute, an interdisciplinary research institute established in 2003 that focuses on equity and inclusion. During this session, we initially watched the implicit bias module together and then had breakout Zoom discussions focusing on our own implicit biases that facilitated by small group leaders. The third session was a lecture-based format focusing on financial toxicity in cancer care, which was led by Dr. Samilia Obeng-Gyasi. The fourth session centered on cancer health equity in clinical trial enrollment, which was led by the executive director for the OSU Center for Cancer Health Equity. We also compiled both institutional and online resources for personal reading and training to supplement the formal curriculum.

#### Curriculum survey and statistical analysis

A pre- and post-curriculum survey was implemented for anonymous feedback of our curriculum. The pre-curriculum survey collected information on demographics, baseline understanding of health care and cancer disparities education in the fellowship program, and potential areas of focus for the curriculum. The areas of understanding assessed were: [1] overall disparities in healthcare and [2] racial/ethnic disparities, [3] socioeconomic disparities, [4] gender-identity and sexual orientation and [5] language barriers in Hematology/Oncology, as well as [6] disparities in clinical trials and access to novel therapies. The post-curriculum survey asked the same understanding questions as the pre-curriculum survey to assess efficacy and usefulness of these sessions. Using a Likert scale (Poor = 1, Fair = 2, Good = 3, Very Good = 4, Excellent = 5) we assessed fellows’ level of agreement with the following statements:: “the presentations were organized,” “the information presented was relevant to my practice,” “overall expectations were met,” “the information presented is useful for training as a hematology/oncologist,” and the discussions improved my insight into disparities in hematology/oncology.” We also assessed the preferred format of sessions for the curriculum, if the fellows need more training in healthcare disparities, if they will incorporate health disparities into future research projects, if they will recommend the curriculum to future OSU hematology/oncology fellows, and if they recommend fellowship programs have formal health disparities curricula. We utilized a mixed methods approach with descriptive statistics and open-ended questions.

## Results

Baseline characteristics of participants are described in Table 1. Fifteen fellows completed the pre-curriculum survey, out of 20 total Hematology/Oncology fellows. Thirteen (86%) of the fellows were ages 25–35 years, and 10 (67%) identified as White or Asian. Of the thirteen responding to the gender question, 7(54%) identified as male, 5(38%) identified as female, and 1 preferred not to answer the question. Fourteen (93%) of the fellows had not previously worked on a health disparities-focused research project.

**Table 1:** Baseline Demographics of Fellows

Number of participants	15
Age (n = 14)	
25–30	4 (28.5)
31–35	9 (64.5)
36–40	1 (7.1)
Gender (n = 13)	
Female	7 (53.9)
Male	5 (38.5)
Prefer not to answer	1 (7.6)
Race (n = 13)	
White	4 (30.8)
Asian	6 (45.1)
Hispanic	1 (7.6)
Mixed	1 (7.6)
Prefer not to answer	1 (7.6)
Previous research experience in healthcare disparities	
Yes	1(6.7)
No	14 (93.3)

### Pre-curriculum survey

**Fig. 1:**
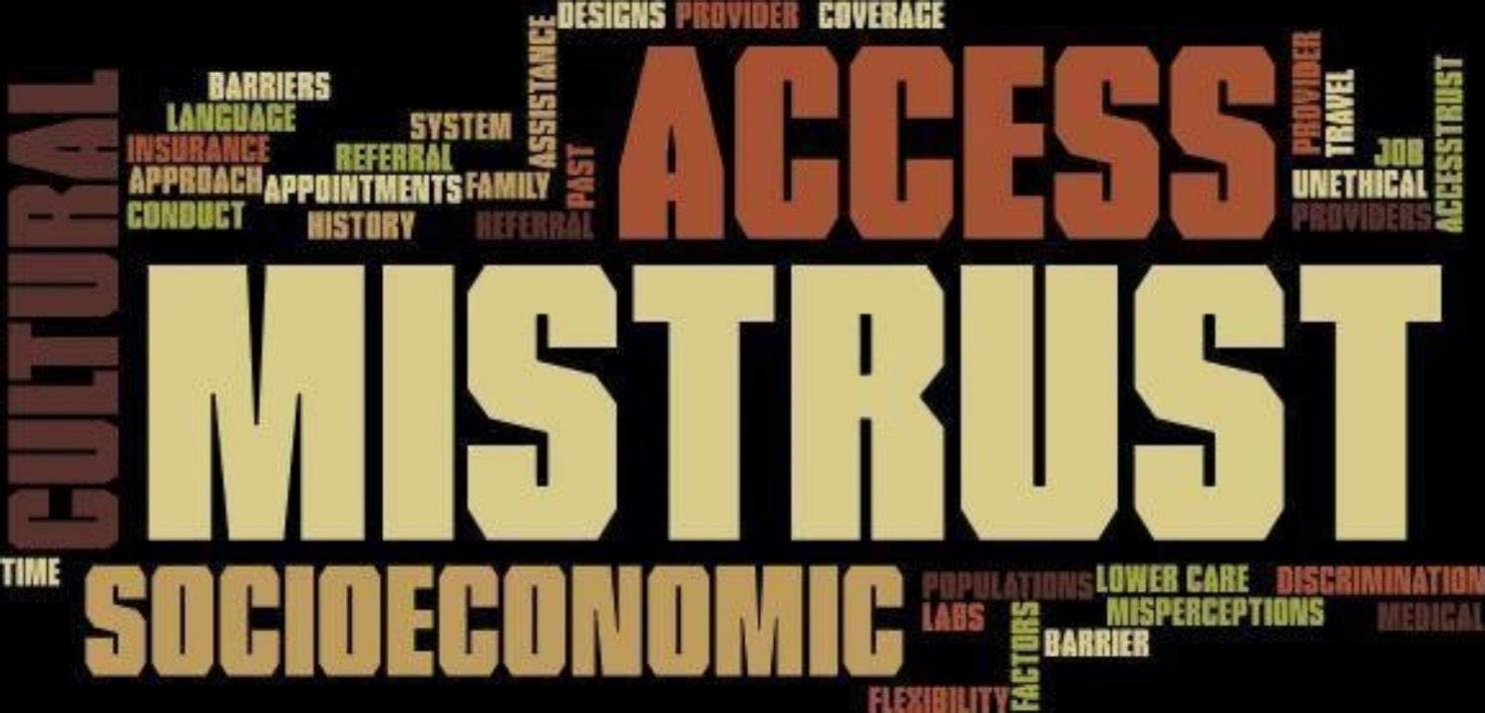
Factors resulting in decreased representation of minorities in clinical trials

The pre-curriculum survey was completed prior to the first session (Table 2). Fellows were asked about their understanding of various health care disparities in hematology and oncology. Twelve fellows (80%) had a fair or good understanding of overall disparities in healthcare. Regarding specific disparities in the field of hematology/oncology: 9 fellows (60%) had a fair or good understanding of racial/ethnic disparities; 8 fellows (53%) had a fair or good understanding of socioeconomic and gender-identity/sexual orientation disparities; 11 fellows (74%) had a fair or good understanding of language barriers; and 10 fellows (67%) had a fair or good understanding of disparities in clinical trials and access to novel therapies. The fellows were asked about their views on factors that can explain decreased representation of minorities in clinical trials. A word cloud was created from the keywords fellows reported. The most common responses were mistrust, socioeconomics and limited access to clinical trials (Fig. 1).

**Table 2:** Results of Pre-Curriculum Survey

PRE-CURRICULUM SURVEY					
n (%)	Poor	Fair	Good	Very Good	Excellent
**Overall clinical training in disparities in healthcare**	0 (0)	6 (40)	6 (40)	1 (6.7)	2 (13.3)
**Understanding of racial/ethical disparities in Hematology/Oncology**	1 (6.7)	3 (20)	6(40)	4(26.7)	1 (6.7)
**Understanding of socioeconomic disparities in Hematology/Oncology**	2 (13)	2 (13)	6 (40)	3 (20)	2 (13)
**Understanding of gender-identity and sexual orientation in Hematology/Oncology**	3 (20)	3 (20)	5 (33.3)	3 (20)	1 (6.7)
**Understanding of language barriers in** **Hematology/Oncology**	0 (0)	4 (26.7)	7 (46.7)	2 (13.3)	2 (13.3)
**Disparities in clinical trials and access to novel therapies**	1 (6.7)	6 (40)	4 (26.7)	4 (26.7)	0 (0)
**The information presented is useful for training as a Hematologist/Oncologist**	0 (0)	0 (0)	2 (14.2)	4 (28.6)	8 (57.1)
**The discussions improved my insight into disparities in Hematology/Oncology**	0 (0)	0 (0)	2 (14.2)	4 (28.6)	8 (57.1)

### Post-curriculum survey

The post-curriculum survey was sent after the final session (Table 3). Fourteen of fifteen participants completed the post-curriculum survey. The mean scores for understanding of the various cancer disparities improved after the implementation curriculum for each area assessed: +0.7 to + 1 (Table 4). Thirteen fellows (93%) noted “agree” or “strongly agree” that the presentations were well organized with 12 fellows (86%) noting overall expectations were met (“agree” or “strongly agree”). Twelve fellows (86%) noted “agree” or “strongly agree” that the information presented was relevant to their practice. Twelve fellows (86%) noted “agree” or “strongly agree” that the information presented is useful for training as a hematologist/oncologist, and that the discussions improved their insight into disparities in the field of hematology/oncology. Eleven fellows (79%) affirmed incorporating healthcare disparities into their future research plans. Ten out of 15 fellows (71%) felt a need for further training in cancer disparities. The majority of fellows, 10 (71%), would recommend the curriculum to other fellows with 12 (80%) noting that their favorite format was the panel discussions. Eleven fellows (79%) noted it would be a good idea to incorporate cancer disparities education into the formal fellowship curriculum. The most common suggestions for future topics were gender identity, homeless and undocumented patients, and health literacy.

**Table 3:** Results of Post-Curriculum Survey

POST-CURRICULUM SURVEY					
	**Strongly** **Disagree**	**Disagree**	**Neutral**	**Agree**	**Strongly Agree**
**The presentations were well organized**	0 (0)	0 (0)	1 (7.1)	6 (42.9)	7 (50)
**The information presented was relevant to my practice**	0 (0)	0 (0)	2 (14.2)	9 (64.3)	3 (21.3)
**Overall expectations were met**	0 (0)	0 (0)	2 (14.2)	4 (28.6)	8 (57.1)
**The information presented is useful for training as a Hematologist/Oncologist**	0 (0)	0 (0)	2 (14.2)	4 (28.6)	8 (57.1)
**The discussions improved my insight into disparities in Hematology/Oncology**	0 (0)	0 (0)	2 (14.2)	4 (28.6)	8 (57.1)
	**Poor**	**Fair**	**Good**	**Very Good**	**Excellent**
**Overall clinical training in disparities in healthcare**	0 (0)	0 (0)	3 (21.4)	9 (64.3)	2 (15)
**Understanding of racial/ethical disparities in Hematology/Oncology**	0 (0)	1 (7.15)	4 (28.6)	6 (42.9)	3 (21.4)
**Understanding of gender-identity and sexual orientation in Hematology/Oncology**	0 (0)	2 (14.3)	4(28.6)	5 (35.7)	3 (21.4)
**Understanding of language barriers in** **Hematology/Oncology**	0 (0)	1 (7.1)	4(28.6)	4(28.6)	5 (35.7)
**Disparities in clinical trials and access to novel therapies**	0 (0)	1 (7.1)	4(28.6)	6 (42.9)	3 (21.4)
	Journal articles	Panel discussions	Modules	Podcasts	
**What was your favorite session or part of the curriculum?**	1 (6.67%)	12 (80%)	1 (6.67%)	1 (6.67%)	
	Yes	No			
**Do you feel you need more** **training in healthcare disparities?**	10 (71%)	4 (29%)			
	Yes	No			
**After this focus on health disparities, do you plan to incorporate health disparities into a future research or clinical project?**	11 (79%)	1 (7%)			
	Yes	No			
**Would you recommend this curriculum to future hematology/oncology fellows?**	10 (71%)	0 (0%)			
	Yes	No			
**Do you recommend that the fellowship program have a formal health disparities curriculum?**	11 (79%)	3 (21%)			

**Table 4:** Comparison of mean scores of understanding disparities among fellows (between the pre- and post-curriculum survey) (Scoring: Poor = 1, Fair = 2, Good = 3, Very Good = 4, Excellent = 5)

	Presurvey		Post survey		Difference
	Mean	SD	Mean	SD	
**Overall clinical training in disparities in healthcare**	2.93	1.00	3.93	0.59	+ 1
**Understanding of racial/ethical disparities in hematology/oncology**	3.07	1.00	3.79	0.86	+ 0.7
**Understanding of gender-identity and sexual orientation in hematology/oncology**	2.73	1.18	3.64	0.97	+ 0.9
**Understanding of language barriers in hematology/oncology**	3.13	0.96	3.93	0.96	+ 0.8
**Disparities in clinical trials and access to novel therapies**	2.73	0.93	3.79	0.86	+ 1

## Discussion

We present the implementation of pilot cancer disparities curriculum for the OSU Hematology/Oncology Fellowship program. We used a structured curriculum of lectures, interactive sessions, and self-learning modules to improve the understanding of cancer disparities amongst fellows. We demonstrated that hematology/oncology fellows did not have a strong baseline understanding of cancer disparities and have not previously participated in a research project focused on health disparities. After implementation of the disparities curriculum, fellows noted an improvement in the understanding of cancer disparities. They noted that overall expectations were met, and the presentations were well organized, improving their insights into disparities in the field of hematology/oncology. Fellows would recommend further training in disparities as part of the formal fellowship curriculum as they noted this disparities curriculum improved their training as a hematologist/oncologist.

The need for educating physicians and medical trainees on healthcare disparities is well-established. The National Academy of Medicine published the seminal report on health disparities amongst minority populations, specifically calling for physician education of health care disparities [12]. Despite the significant attention the report received, a follow-up study in 2018 found that healthcare disparities still existed among different minority groups and were most prevalent in patients who are low-income and uninsured [13]. Previous studies focusing on residents of various medical specialties have demonstrated that trainees report inadequate training in addressing healthcare disparities, particularly religious beliefs, immigrant health and belief systems affecting healthcare, while noting confidence in treating the medical issues of their patients [14]. In a survey of residents treating patients with cancer, 29% of respondents did not understand the socioeconomic background of their patients [15]. Similarly, a survey of more than 20,000 residents across 227 internal medicine residency programs demonstrated that only 39% of programs employed a disparities curriculum [16]. There is limited data on the development of standardized curriculum on cancer disparities for advanced trainees, particularly for hematology/oncology fellows.

Our curriculum consisted of lectures, self-learning modules, and panel discussions (see Table 5 more information). We implemented diverse learning formats to accommodate different learning styles. Of all the formats, 12 fellows (80%) favored panel discussions. Overall, our curriculum increased the understanding of disparities in cancer care and 11 fellows (78%) will consider a research project in cancer care disparities in the future.

**Table 5:** Detailed information of the pilot curriculum

Format	Title/Focus area	Description
Lectures		
	Healthcare disparities in hematology/oncology: disparities in care related to sickle cell disease, multiple myeloma, and breast cancer	Panel discussion
	Implicit bias: Modules provided by the OSU Kirwan Institute, an interdisciplinary research institute established in 2003 that focuses on equity and inclusion	Module viewing followed by discussion
	Financial Toxicity	Journal club
	Cancer health equity in clinical trial enrollment	Panel discussion
Modules and Videos		
	American Society of Clinical Oncology (ASCO) modules	Cultural Literacy
	American Academy of Family Physicians (AAFP) Health Equity Curricular Toolkit	Curriculum guide
	Race and Racism	
	“The Danger of a Single Story”	
	BuckeyeLearn	Institution-specific modules on diversity training for faculty and staff
	OSU Kirwan Institute for the Study of Race and Ethnicity	Trainings on Implicit Bias
Lectures/Workshops		
	Implicit bias workshop by Dr. Capers	Institution professor discussing implicit bias impact on chronic disease
	Center for Cancer Health Equity	Lunch & Learn webinar series
Podcasts		
	Outspoken Oncology	Discussion on the most pressing—and often controversial—topics in cancer care today
Additional online resources		
	AMA Health Equity language, Narrative and Concepts	Health equity guide, and for the AMA
	How to classify racial/ethnic groups	Editorial
	Disparities framework	Theoretical and Methodological Gap within Environmental Justice Research

There were several key limitations with our curriculum. This was a pilot project and we did not perform a formal intervention analysis. We provided self-learning modules that were voluntary and we did not track their completion. The virtual format due to the COVID-19 pandemic also limited the didactic interactions between the presenter and the participants.

Given the initial success of this pilot project, we plan to incorporate this cancer disparities curriculum into the formal fellowship program and are currently working on optimizing the curriculum and topics based on fellow feedback. While our pilot curriculum was voluntary, in the future we plan to formally implement it into the fellowship program so that it benefit all fellows. We hope this curriculum can help inform other hematology/oncology fellowships that are working on implementing or optimizing existing healthcare disparities curricula.

## Conclusions

We incorporated a pilot cancer disparities didactic curriculum into a hematology/oncology fellowship program. The majority of fellows offered this curriculum participated in its sessions and requested ongoing education about healthcare disparities.

## Data Availability

All data generated or analyzed during this study are included in this published article. Please contact Dr. Marium Husain (corresponding author) if data from this study is requested.
